# Validity and significance of 30‐day mortality rate as a quality indicator for gastrointestinal cancer surgeries

**DOI:** 10.1002/ags3.12070

**Published:** 2018-04-16

**Authors:** Tsunekazu Mizushima, Hiroyuki Yamamoto, Shigeru Marubashi, Kinji Kamiya, Go Wakabayashi, Hiroaki Miyata, Yasuyuki Seto, Yuichiro Doki, Masaki Mori

**Affiliations:** ^1^ Department of Surgery Gastroenterological Surgery Graduate School of Medicine Osaka University Osaka Japan; ^2^ Department of Healthcare Quality Assessment Graduate School of Medicine The University of Tokyo Tokyo Japan; ^3^ Department of Regenerative Surgery Fukushima Medical University Fukushima Japan; ^4^ Second Department of Surgery Hamamatsu University School of Medicine Hamamatsu Japan; ^5^ Database Committee The Japanese Society of Gastroenterological Surgery Tokyo Japan; ^6^ The Japanese Society of Gastroenterological Surgery Tokyo Japan

**Keywords:** 30‐day mortality rate, gastrointestinal cancer, hepatobiliary cancer, pancreatic cancer, quality indicator

## Abstract

**Background and Aim:**

Benchmarking has proven beneficial in improving the quality of surgery. Mortality rate is an objective indicator, of which the 30‐day mortality rate is the most widely used. However, as a result of recent advances in medical care, the 30‐day mortality rate may not cover overall surgery‐related mortalities. We examined the significance and validity of the 30‐day mortality rate as a quality indicator.

**Methods:**

The present study was conducted on cancer surgeries of esophagectomy, total gastrectomy, distal gastrectomy, right hemicolectomy, low anterior resection, hepatectomy, and pancreaticoduodenectomy that were registered in the first halves of 2012, 2013 and 2014 in a Japanese nationwide large‐scale database. This study examined the mortality curve for each surgical procedure, “sensitivity of surgery‐related death” (capture ratio) at each time point between days 30‐180, and the association between mortality within 30 days, mortality after 31 days, and preoperative, perioperative, and postoperative factors.

**Results:**

Surgery‐related mortality rates of each surgical procedure were 0.6%‐3.0%. Regarding 30‐day mortality rates, only 38.7% (esophagectomy) to 53.3% (right hemicolectomy) of surgery‐related mortalities were captured. The capture ratio of surgery‐related deaths reached 90% or higher for 120‐day to 150‐day mortality rates. Factors associated with mortality rate within 30 days/after the 31st day were different, depending on the type of surgical procedure.

**Conclusion:**

Thirty‐day mortality rate is useful as a quality indicator, but is not necessarily sufficient for all surgical procedures. Quality of surgery may require evaluation by combining 30‐day mortality rates with other indicators, depending on the surgical procedure.

## 1 INTRODUCTION

Benchmarking and evaluations of surgical quality have proven beneficial and indispensable for improving surgical quality.^1^, ^2^ A technique that focuses on the “structure,” “process,” “results,” and “outcomes” of medical care has been proposed as a method for evaluating the quality of medical care, and a variety of other parameters are also being used as indicators in the assessment of surgical quality.^3^ For example, number of surgeries has been used as a “structural” indicator, rate of laparoscopic surgeries and length of hospital stay have been used as “process” indicators, and surgery‐related mortality rate, as well as the rate of complications, has been used as “outcome” indicators. In recent years, interest has been increasingly shown in, as well as expectations from, outcome‐oriented evaluations and measurements of medical care quality.

Mortality is an objective indicator, and assessment methods using overall mortality, surgery‐related mortality, in‐hospital mortality, 30‐day mortality, and 90‐day mortality are commonly used. Historically, the 30‐day mortality rate has been used to measure performance across a wide range of surgical disciplines, and the American College of Surgeons National Surgical Quality Improvement Program (ACSNSQIP) database, which is widely considered as the gold standard database for surgical quality improvements and future pay‐for‐performance programs, records 30‐day complication and mortality outcomes after surgery.^4^, ^5^, ^6^ The 30‐day mortality rate, as an objective indicator, may not entirely encompass the overall surgery‐related mortality rate.^7^, ^8^ As a result of recent progress in medical care, especially advances in anaesthesia^9^ and intensive care,^10^ early postoperative mortality rates have decreased and survival rates have improved among patients who could have died early after surgery if treated using previous methods. However, this does not necessarily mean that all patients who avoid death during the early postoperative period will continue to survive. Medical circumstances, such as length of hospital stay after surgery, are different in each country, and patients particularly in Europe and the USA are discharged from hospitals during the early postoperative period. As a result, surgery‐related complications that occur after discharge might be impossible to determine on the basis of 30‐day mortality rates or in‐hospital mortality rates. There is an absence of available data pertaining to how much of the surgery‐related mortality rate can be determined based on the 30‐day mortality rate, although this observation varies depending on situations in each country.

Herein, our study examines the significance and validity of the 30‐day mortality rate as a surgery‐related quality indicator (QI).

## 2 METHODS

### 2.1 Data collection

The National Clinical Database (NCD) was established in 2010 as a nationwide database that registers all surgical cases in cooperation with the surgical board certification system. Registrations started in 2011 and, currently, approximately 4300 facilities all over Japan are participating in the registry, and approximately 97% of surgical operations carried out by surgeons have been registered.^11^ The NCD, a Web‐based data management system, continuously involves individuals who approve data, those in charge of annual case reports from various departments, and data entry personnel, thereby assuring data traceability. Among gastrointestinal surgeries registered in the NCD, data on items similar to those of the ACSNSQIP have been collected in regard to esophagectomy, total gastrectomy, distal gastrectomy, right hemicolectomy, low anterior resection, hepatectomy, pancreaticoduodenectomy, and acute diffuse peritonitis. Hepatectomy included only extended lobectomy, lobectomy and segmentectomy other than lateral segmentectomy for primary liver cancer or gallbladder cancer.

NCD records between January 1, 2012 and December 31, 2014 were analyzed for this study. The study was conducted on cases of gastrointestinal cancers, which were treated with esophagectomy, total gastrectomy, distal gastrectomy, right hemicolectomy, low anterior resection, hepatectomy, and pancreaticoduodenectomy. To examine the significance and validity of the 30‐day mortality rate as a surgical QI, the study was carried out without including acute diffuse peritonitis, in which the patient's condition before surgery may have a great influence on the mortality rate. In addition, cases of non‐curative resection were excluded to exclude cancer‐related deaths. In order to increase the quality of the endpoints, a longer duration was given to the postoperative observation period, and data on surgical cases operated between January and June, which allowed for acquisition of follow‐up data from 30 days to 180 days after surgery, were analyzed. Records from patients who refused use of their data were excluded from this analysis. Records with missing data for age, gender, or status at postoperative day 30 were also excluded. Because non‐curative resection cases had been excluded, postoperative mortality was equivalent to surgery‐related mortality and, therefore, was considered as an endpoint.

### 2.2 Mortality curve for each operative procedure

Mortality curves were drawn using death within 30 days or postoperative death as events to visualize when mortality events occurred in the postoperative course.

### 2.3 Sensitivity of surgery‐related mortality (capture ratio)

In order to assess the mortality at various time points, ranging from 30 days to 180 days, which covers the surgery‐related mortality rate, we calculated the sensitivity of mortality at each point (capture ratio) on the basis of the 210‐day mortality, and we also calculated respective 95% confidence intervals.

### 2.4 Association between mortality within 30 days, mortality after 31 days, and preoperative, perioperative, and postoperative factors

Preoperative, perioperative, and postoperative factors, extracted during the creation of surgery‐related mortality models, were used for examining association of mortality within 30 days and after 31 days. We investigated the distribution of patients who died within 30 days as well as that of patients who died after the 31st day, and the difference between the two was tested using Fisher's exact test. In both categories, a two‐sided *P*‐value of <.05 was considered statistically significant. Statistical analyses were carried out using STATA14 (Stata Corp., College Station, TX, USA).

The present study followed the ethical guidelines of human subjects based on the Helsinki Declaration. Review and approval by the ethics committee was not carried out because existing unlinkable, anonymized data were used in the present study.

## 3 RESULTS

### 3.1 Subjects

Our study included 7448 cases of esophagectomy, 22 453 cases of total gastrectomy, 48 774 cases of distal gastrectomy, 24 260 cases of right hemicolectomy, 27 046 cases of low anterior resection, 7486 cases of hepatectomy, and 10 550 cases of pancreaticoduodenectomy. Among patients with esophagectomies, 39.5% presented a history of smoking tobacco within 1 year before surgery, and 62.0% had an alcohol‐consumption habit before surgery. Among patients with hepatectomies and those with pancreaticoduodenectomies, patients who were diagnosed with diabetes mellitus before surgery accounted for 28.1% and 30.2%, respectively, and these rates were higher than those found in patients undergoing other surgical procedures. Patients with intraoperative blood loss of 1000 mL or more accounted for 37.9% of those treated for hepatectomies, and 34.2% of those treated for pancreaticoduodenectomies, and patients with postoperative complications of Clavien‐Dindo classification grade III or greater accounted for 16.9% of those treated for esophagectomies and 16.0% of those treated for pancreaticoduodenectomies. Patients with postoperative anastomotic leakage accounted for 11.6% of those treated for esophagectomies, 8.5% of those treated for low anterior resections, and 10.6% of those treated for pancreaticoduodenectomies. Finally, 20.7% of patients treated for pancreaticoduodenectomies developed pancreatic fistulas (any grade), 2.3% developed grade C pancreatic fistulas, and bile leakage was present in 6.7% of patients treated for hepatectomies (Table 1).

**Table 1:** Characteristics of analyzed subjects

		Esophagectomy (n = 7448)		Total gastrectomy (n = 22 453)		Distal gastrectomy (n = 48 774)		Right hemicolectomy (n = 24 260)		Low anterior resection (n = 27 046)		Hepatectomy (n = 7486)		Pancreaticoduodenectomy (n = 10 550)		Total (n = 148 017)	
		No.	%	No.	%	No.	%	No.	%	No.	%	No.	%	No.	%	No.	%
Age (y)	‐59	1387	18.6	3340	14.9	7953	16.3	2569	10.6	6085	22.5	989	13.2	1372	13.0	23 695	16.0
	60‐64	1477	19.8	3148	14.0	7005	14.4	2377	9.8	4587	17.0	1056	14.1	1474	14.0	21 124	14.3
	65‐69	1671	22.4	3652	16.3	7675	15.7	3247	13.4	4806	17.8	1305	17.4	1983	18.8	24 339	16.4
	70‐74	1586	21.3	4553	20.3	8877	18.2	4464	18.4	4698	17.4	1618	21.6	2361	22.4	28 157	19.0
	75‐79	947	12.7	4207	18.7	8339	17.1	4732	19.5	3607	13.3	1659	22.2	2122	20.1	25 613	17.3
	80‐	380	5.1	3553	15.8	8925	18.3	6871	28.3	3263	12.1	859	11.5	1238	11.7	25 089	17.0
Gender	Female	1223	16.4	5701	25.4	16 204	33.2	12 450	51.3	9442	34.9	2042	27.3	4164	39.5	51 226	34.6
	Male	6225	83.6	16 752	74.6	32 570	66.8	11 810	48.7	17 604	65.1	5444	72.7	6386	60.5	96 791	65.4
Smoking habits	(‐)	4509	60.5	17 534	78.1	38 700	79.3	21 378	88.1	21 410	79.2	6077	81.2	8657	82.1	118 265	79.9
	(+)	2939	39.5	4919	21.9	10 074	20.7	2882	11.9	5636	20.8	1409	18.8	1893	17.9	29 752	20.1
Drinking habits	(‐)	2830	38.0	16 000	71.3	35 455	72.7	20 008	82.5	19 739	73.0	5477	73.2	7950	75.4	107 459	72.6
	(+)	4618	62.0	6453	28.7	13 319	27.3	4252	17.5	7307	27.0	2009	26.8	2600	24.6	40 558	27.4
Hypertension	(‐)	5033	67.6	14 128	62.9	30 376	62.3	14 715	60.7	17 790	65.8	4407	58.9	6595	62.5	93 044	62.9
	(+)	2415	32.4	8325	37.1	18 398	37.7	9545	39.3	9256	34.2	3079	41.1	3955	37.5	54 973	37.1
Diabetes	(‐)	6507	87.4	18 488	82.3	40 319	82.7	19 876	81.9	22 305	82.5	5385	71.9	7369	69.8	120 249	81.2
	(+)	941	12.6	3965	17.7	8455	17.3	4384	18.1	4741	17.5	2101	28.1	3181	30.2	27 768	18.8
Chronic obstructive pulmonary disease	(‐)	6945	93.2	21 405	95.3	46 734	95.8	23 568	97.1	26 221	96.9	7219	96.4	10 253	97.2	142 345	96.2
	(+)	503	6.8	1048	4.7	2040	4.2	692	2.9	825	3.1	267	3.6	297	2.8	5672	3.8
Brain disorder	(‐)	7273	97.7	21 610	96.2	46 946	96.3	23 295	96.0	26 181	96.8	7273	97.2	10 214	96.8	142 792	96.5
	(+)	175	2.3	843	3.8	1828	3.7	965	4.0	865	3.2	213	2.8	336	3.2	5225	3.5
≥ASA2	(‐)	2418	32.5	6986	31.1	16 262	33.3	6649	27.4	9829	36.3	1732	23.1	2678	25.4	46 554	31.5
	(+)	5030	67.5	15 467	68.9	32 512	66.7	17 611	72.6	17 217	63.7	5754	76.9	7872	74.6	101 463	68.5
Intraoperative cardiac complications	(‐)	7438	99.9	22 443	100.0	48 755	100.0	24 251	100.0	27 037	100.0	7481	99.9	10 547	100.0	14 7952	100.0
	(+)	10	0.1	10	0.0	19	0.0	9	0.0	9	0.0	5	0.1	3	0.0	65	0.0
Intraoperative blood loss ≥1000 mL	(‐)	6709	90.1	20 261	90.2	47 436	97.3	23 718	97.8	25 721	95.1	4649	62.1	6938	65.8	135 432	91.5
	(+)	739	9.9	2192	9.8	1338	2.7	542	2.2	1325	4.9	2837	37.9	3612	34.2	12 585	8.5
Postoperative complications Clavien‐Dindo classification ≥grade III	(‐)	6188	83.1	20 642	91.9	46 507	95.4	23 195	95.6	24 612	91.0	6595	88.1	8867	84.0	136 606	92.3
	(+)	1260	16.9	1811	8.1	2267	4.6	1065	4.4	2434	9.0	891	11.9	1683	16.0	11 411	7.7
Postoperative complications Clavien‐Dindo classification ≥grade IV	(‐)	7199	96.7	22 118	98.5	48 322	99.1	24 044	99.1	26 782	99.0	7268	97.1	10 251	97.2	145 984	98.6
	(+)	249	3.3	335	1.5	452	0.9	216	0.9	264	1.0	218	2.9	299	2.8	2033	1.4
Reoperation within 30 days after operation	(‐)	6768	90.9	21 332	95.0	47 301	97.0	23 414	96.5	24 654	91.2	7218	96.4	9882	93.7	140 569	95.0
	(+)	680	9.1	1121	5.0	1473	3.0	846	3.5	2392	8.8	268	3.6	668	6.3	7448	5.0
Surgical site infection other than anastomotic leakage	(‐)	6828	91.7	21 304	94.9	47 406	97.2	23 016	94.9	25 835	95.5	7042	94.1	9458	89.6	140 889	95.2
	(+)	620	8.3	1149	5.1	1368	2.8	1244	5.1	1211	4.5	444	5.9	1092	10.4	7128	4.8
Anastomotic leakage	(‐)	6584	88.4	21 524	95.9	47 749	97.9	23 929	98.6	24 741	91.5	7373	98.5	9432	89.4	141 332	95.5
	(+)	864	11.6	929	4.1	1025	2.1	331	1.4	2305	8.5	113	1.5	1118	10.6	6685	4.5
Transfusion ≥5 U	(‐)	7279	97.7	22 234	99.0	48 502	99.4	24 144	99.5	26 914	99.5	7224	96.5	10 220	96.9	146 517	99.0
	(+)	169	2.3	219	1.0	272	0.6	116	0.5	132	0.5	262	3.5	330	3.1	1500	1.0
Postoperative unexpected intubation	(‐)	7012	94.1	22 165	98.7	48 414	99.3	24 150	99.5	26 909	99.5	7353	98.2	10 334	98.0	146 337	98.9
	(+)	436	5.9	288	1.3	360	0.7	110	0.5	137	0.5	133	1.8	216	2.0	1680	1.1
Postoperative mechanical ventilation ≥48 h	(‐)	6881	92.4	22 157	98.7	48 418	99.3	24 099	99.3	26 878	99.4	7332	97.9	10 313	97.8	146 078	98.7
	(+)	567	7.6	296	1.3	356	0.7	161	0.7	168	0.6	154	2.1	237	2.2	1939	1.3
Postoperative renal dysfunction	(‐)	7318	98.3	22 264	99.2	48 493	99.4	24 121	99.4	26 852	99.3	7331	97.9	10 378	98.4	146 757	99.1
	(+)	130	1.7	189	0.8	281	0.6	139	0.6	194	0.7	155	2.1	172	1.6	1260	0.9
Postoperative central nervous system disorder	(‐)	7430	99.8	22 400	99.8	48 689	99.8	24 215	99.8	26 998	99.8	7476	99.9	10 528	99.8	147 736	99.8
	(+)	18	0.2	53	0.2	85	0.2	45	0.2	48	0.2	10	0.1	22	0.2	281	0.2
Postoperative cardiac complications	(‐)	7389	99.2	22 360	99.6	48 653	99.8	24 203	99.8	26 981	99.8	7445	99.5	10 478	99.3	147 509	99.7
	(+)	59	0.8	93	0.4	121	0.2	57	0.2	65	0.2	41	0.5	72	0.7	508	0.3
Postoperative sepsis	(‐)	7066	94.9	21 954	97.8	48 198	98.8	23 978	98.8	26 509	98.0	7299	97.5	10 096	95.7	145 100	98.0
	(+)	382	5.1	499	2.2	576	1.2	282	1.2	537	2.0	187	2.5	454	4.3	2917	2.0
Postoperative septic shock	(‐)	7364	98.9	22 324	99.4	48 618	99.7	24 156	99.6	26 921	99.5	7438	99.4	10 447	99.0	147 268	99.5
	(+)	84	1.1	129	0.6	156	0.3	104	0.4	125	0.5	48	0.6	103	1.0	749	0.5
Postoperative pneumonia	(‐)	6543	87.8	21 783	97.0	47 884	98.2	23 991	98.9	26 844	99.3	7364	98.4	10 316	97.8	144 725	97.8
	(+)	905	12.2	670	3.0	890	1.8	269	1.1	202	0.7	122	1.6	234	2.2	3292	2.2
Postoperative deep vein thrombosis	(‐)	7413	99.5	22 415	99.8	48 714	99.9	24 212	99.8	26 983	99.8	7460	99.7	10 511	99.6	147 708	99.8
	(+)	35	0.5	38	0.2	60	0.1	48	0.2	63	0.2	26	0.3	39	0.4	309	0.2
Postoperative pulmonary embolism	(‐)	7428	99.7	22 429	99.9	48 727	99.9	24 244	99.9	27 011	99.9	7465	99.7	10 521	99.7	147 825	99.9
	(+)	20	0.3	24	0.1	47	0.1	16	0.1	35	0.1	21	0.3	29	0.3	192	0.1
Postoperative urinary tract infection	(‐)	7406	99.4	22 353	99.6	48 589	99.6	24 134	99.5	26 783	99.0	7440	99.4	10 471	99.3	147 176	99.4
	(+)	42	0.6	100	0.4	185	0.4	126	0.5	263	1.0	46	0.6	79	0.7	841	0.6
Postoperative pancreatic fistula	(‐)			21 394	95.3	47 929	98.3							8363	79.3	77 686	95.0
	(+)			1059	4.7	845	1.7							2187	20.7	4091	5.0
Postoperative pancreatic fistula ≥grade C	(‐)			22 382	99.7	48 706	99.9							10 304	97.7	81 392	99.5
	(+)			71	0.3	68	0.1							246	2.3	385	0.5
Postoperative biliary fistula	(‐)											6981	93.3			6981	93.3
	(+)											505	6.7			505	6.7

ASA2, American Society of Anesthesiologists Classification 2.

### 3.2 Mortality curves for each surgical procedure

When survival or death at day 210 was used as an endpoint, the surgery‐related mortality rate was 3.0% for patients with hepatectomies, 2.5% for patients with pancreaticoduodenectomies, 2.3% for patients with esophagectomies, 1.4% for patients with total gastrectomies, 1.1% for patients with right hemicolectomies, 0.8% for patients with distal gastrectomies, and 0.6% for patients with low anterior resections, respectively, in descending order. All types of surgical procedures showed mortality rates that increased over time.

**Figure 1:**
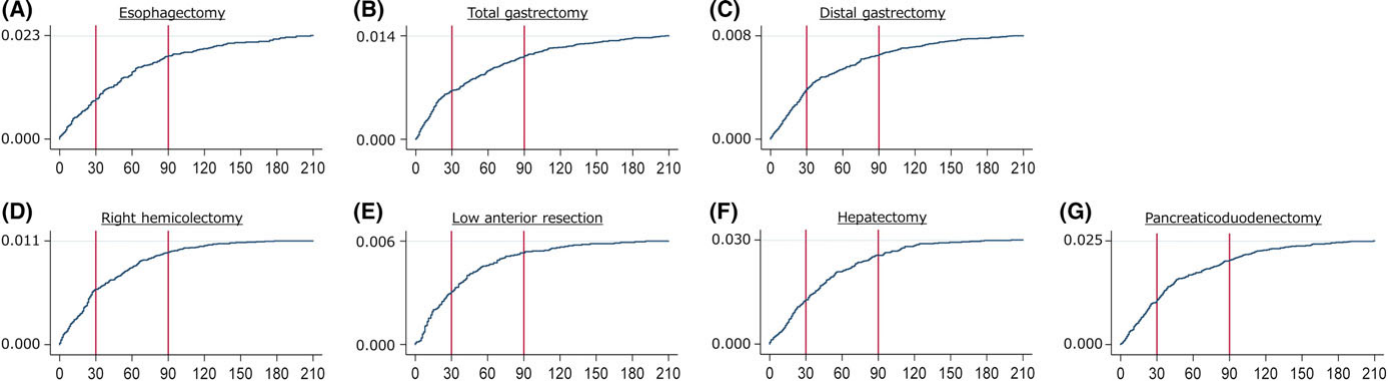
Mortality curve for each type of surgical procedure. A, Esophagectomy; B, Total gastrectomy; C, Distal gastrectomy; D, Right hemicolectomy; E, Low anterior resection; F, Hepatectomy; G, Pancreaticoduodenectomy

For right hemicolectomies, low anterior resections, and hepatectomies, the mortality rate nearly reached a plateau between days 90 and 150. For esophagectomies, total gastrectomies, gastrectomies, and pancreaticoduodenectomies, the mortality rate continuously increased until day 210 (Figure 1).

### 3.3 Sensitivity of the 30‐to‐180‐day mortality rate (capture ratio for surgery‐related mortalities) in comparison with the 210‐day mortality rate (surgery‐related mortalities)

The 30‐day mortality rate captured only 38.7% (esophagectomies) to 53.3% (right hemicolectomies) of surgery‐related mortalities. The capture ratio for surgery‐related mortalities reached 90% or greater when the 120‐day mortality rate was taken into consideration for right hemicolectomy, low anterior resection, hepatectomy, and pancreaticoduodenectomy, as well as when the 150‐day mortality rate was taken into account for esophagectomy, total gastrectomy, and distal gastrectomy. The capture ratio for surgery‐related mortalities reached 90% or greater when the 120‐day mortality rate was taken into consideration for right hemicolectomy, low anterior resection, hepatectomy, and pancreaticoduodenectomy, and when the 150‐day mortality rate was taken into consideration for esophagectomy, total gastrectomy and distal gastrectomy. The capture ratio of 90‐day mortality rate was 80.3% in esophagectomies, 80.3% in total gastrectomies, 81.6% in distal gastrectomies, 89.0% in right hemicolectomies, 88.5% in low anterior resections, 85.2% in hepatectomies and 81.2% in pancreaticoduodenectomies (Table 2).

**Table 2:** Sensitivity of the 30‐to‐180‐day mortality rate (capture ratio for surgery‐related mortalities)

	30 d	60 d	90 d	120 d	150 d	180 d
Esophagectomy	38.7 (31.4‐46.4)	64.7 (57.1‐71.8)	80.3 (73.6‐85.9)	87.2 (81.3‐91.8)	93.0 (88.1‐96.3)	96.5 (92.6‐98.7)
Total gastrectomy	47.3 (41.7‐52.9)	66.3 (60.8‐71.5)	80.3 (75.6‐84.5)	88.7 (84.8‐92.0)	93.4 (90.1‐95.9)	97.8 (95.6‐99.1)
Distal gastrectomy	48.3 (43.4‐53.2)	68.1 (63.3‐72.5)	81.6 (77.5‐85.2)	89.3 (85.9‐92.1)	95.1 (92.6‐97.0)	97.6 (95.6‐98.8)
Right hemicolectomy	53.3 (47.1‐59.4)	74.4 (68.7‐79.5)	89.0 (84.7‐92.5)	95.4 (92.2‐97.6)	98.4 (96.1‐99.5)	100.0 (98.6‐100.0)
Low anterior resection	50.9 (42.8‐59.0)	76.4 (69.0‐82.8)	88.5 (82.4‐93.0)	94.2 (89.3‐97.3)	97.4 (93.6‐99.3)	98.7 (95.5‐99.8)
Hepatectomy	42.6 (36.0‐49.3)	69.9 (63.4‐75.8)	85.2 (79.8‐89.5)	94.6 (90.7‐97.1)	97.7 (94.8‐99.2)	99.6 (97.5‐100.0)
Pancreaticoduodenectomy	42.9 (36.9‐49.0)	68.2 (62.3‐73.8)	81.3 (76.1‐85.8)	91.0 (86.9‐94.1)	95.1 (91.8‐97.3)	98.5 (96.2‐99.6)

### 3.4 Association between mortality within 30 days, mortality after 31 days, and preoperative, perioperative and postoperative factors

Among factors that likely affect surgery‐related mortality rates, the following showed a significantly higher percentage of deaths within 30 days, irrespective of the type of surgical procedure: postoperative complications of Clavien‐Dindo classification grade III or higher (except esophagectomy), postoperative complications of Clavien‐Dindo classification grade IV or higher (all surgical procedures), unplanned postoperative intubation (except esophagectomy and hepatectomy), and cardiac complications (all surgical procedures). In addition, the following factors were associated with individual surgical procedures: renal dysfunction (distal gastrectomy, right hemicolectomy), central nervous system disorder (right hemicolectomy), sepsis (right hemicolectomy), and septic shock (total gastrectomy, low anterior resection). Conversely, the following factors displayed significantly lower percentages in terms of mortality within 30 days: reoperation within 30 days (esophagectomy, total gastrectomy, right hemicolectomy, pancreaticoduodenectomy), anastomotic leakage (total gastrectomy, distal gastrectomy), pulmonary embolism (low anterior resection), pneumonia (hepatectomy), and urinary tract infection (total gastrectomy, right hemicolectomy) (Table 3).

**Table 3:** Association between mortality within 30 d, mortality after 31 d, and preoperative, perioperative and postoperative factors

	Esophagectomy			Total gastrectomy			Distal gastrectomy			Right colectomy			Low anterior resection			Hepatectomy			Pancreaticoduodenectomy		
	Postoperative death within 30 days (n = 67)	Postoperative death after 31st day (n = 106)	*P*‐value	Postoperative death within 30 days (n = 152)	Postoperative death after 31st day (n = 169)	*P*‐value	Postoperative death within 30 days (n = 152)	Postoperative death after 31st day (n = 169)	*P*‐value	Postoperative death within 30 days (n = 152)	Postoperative death after 31st day (n = 169)	*P*‐value	Postoperative death within 30 days (n = 152)	Postoperative death after 31st day (n = 169)	*P*‐value	Postoperative death within 30 days (n = 152)	Postoperative death after 31st day (n = 169)	*P*‐value	Postoperative death within 30 days (n = 152)	Postoperative death after 31st day (n = 169)	*P*‐value
Age (‐59/60‐64/65‐69/70‐74/75‐79/80‐)	4/14/8/17/18/6	8/17/21/25/24/11	.763	4/11/13/21/48/55	6/10/21/20/47/65	.811	11/10/18/88/38/90	5/6/15/30/52/106	.251	7/6/10/16/25/78	2/3/5/9/19/86	.203	7/9/11/13/12/28	1/9/7/7/16/37	.113	3/14/14/29/24/11	8/21/19/24/36/20	.419	6/13/22/25/23/26	8/10/23/37/39/36	.635
Gender (F/M)	6/61	7/99	.568	26/126	31/138	.884	52/148	56/158	1.000	63/79	60/64	.539	17/63	15/62	.844	21/74	30/98	.873	27/88	36/117	1.000
Smoking habits (+)	36 (53.7%)	48 (45.3%)	.349	**19 (12.5%)**	**36 (21.3%)**	**.039**	31 (15.5%)	29 (13.6%)	.580	9 (6.3%)	14 (11.3%)	.190	19 (23.8%)	13 (16.9%)	.325	20 (21.1%)	32 (25.0%)	.525	25 (21.7%)	22 (14.4%)	.144
Drinking habits (+)	39 (58.2%)	67 (63.2%)	.526	30 (19.7%)	42 (24.9%)	.287	31 (15.5%)	47 (22.0%)	.103	17 (12.0%)	16 (12.9%)	.854	17 (21.3%)	14 (18.2%)	.691	22 (23.2%)	39 (30.5%)	.288	27 (23.5%)	36 (23.5%)	1.000
Hypertension (+)	32 (47.8%)	41 (38.7%)	.270	63 (41.4%)	73 (43.2%)	.821	97 (48.5%)	88 (41.1%)	.139	60 (42.3%)	61 (49.2%)	.269	31 (38.8%)	30 (39.0%)	1.000	42 (44.2%)	61 (47.7%)	.684	63 (54.8%)	80 (52.3%)	.712
Diabetes (+)	16 (23.9%)	20 (18.9%)	.447	24 (15.8%)	14 (8.3%)	.053	49 (24.5%)	52 (24.3%)	1.000	26 (18.3%)	28 (22.6%)	.446	20 (25.0%)	21.0 (27.3%)	.856	26 (27.4%)	43 (33.6%)	.380	43 (37.4%)	60 (39.2%)	.800
Chronic obstructive pulmonary disease (+)	8 (11.9%)	13 (12.3%)	1.000	15 (9.9%)	14 (8.3 %)	.698	21 (10.5%)	21 (9.8%)	.871	9 (6.3%)	10 (8.1%)	.638	5 (6.3%)	3 (3.9%)	.720	4 (4.2%)	7 (5.5%)	.762	10 (8.7%)	8 (5.2%)	.326
Brain disorder (+)	3 (4.5%)	4 (3.8%)	1.000	24 (15.8%)	16 (9.5%)	.093	21 (10.5%)	19 (8.9%)	.620	14 (9.9%)	9 (7.3%)	.516	12 (15.0%)	9 (11.7%)	.641	5 (5.3%)	7 (5.5%)	1.000	8 (7.0%)	14 (9.2%)	.654
≥ASA2	57 (85.1%)	89 (84.0%)	1.000	130 (85.5%)	138 (81.7%)	.370	184 (92.0%)	194 (90.7%)	.728	131 (92.3%)	119 (96.0%)	.301	68 (85.0%)	65 (84.4%)	1.000	84 (88.4%)	109 (85.2%)	.554	102 (88.7%)	134 (87.6%)	.850
Intraoperative cardiac complications (+)	2 (3.0%)	0 (0.0%)	.149	2 (1.3%)	0 (0.0 %)	.223	2 (1.0%)	0 (0.0%)	.233	3 (2.1%)	0 (0.0%)	.251	1 (1.3%)	0 (0.0%)	1.000	1 (1.1%)	0 (0.0%)	.426	0 (0.0%)	0 (0.0%)	
Intraoperative blood loss ≥1000 mL	18 (26.9%)	27 (25.5%)	.860	32 (21.1%)	46 (27.2%)	.241	18 (9.0%)	32 (15.0%)	.071	11 (7.7%)	17 (13.7%)	.160	12 (15.0%)	12 (15.6%)	1.000	65 (68.4%)	88 (68.8%)	1.000	70 (60.9%)	81 (52.9%)	.215
Postoperative complications Clavien‐Dindo classification ≥grade III (+)	55 (82.1%)	79 (74.5%)	.268	**131 (86.2%)**	**103 (60.9%)**	**<.001**	**159 (79.5%)**	**118 (55.1%)**	**<.001**	**113 (79.6%)**	**57 (46.0%)**	**<.001**	**66 (82.5%)**	**44 (57.1%)**	**<.001**	**88 (92.6%)**	**90 (70.3%)**	**<.001**	**104 (90.4%)**	**107 (69.9%)**	**<.001**
Postoperative complications Clavien‐Dindo classification ≥grade IV (+)	**55 (82.1%)**	**51 (48.1%)**	**<.001**	**127 (83.6%)**	**64 (37.9%)**	**<.001**	**148 (74.0%)**	**78 (36.4%)**	**<.001**	**108 (76.1%)**	**29 (23.4%)**	**<.001**	**63 (78.8%)**	**26 (33.8%)**	**<.001**	**87 (91.6%)**	**63 (49.2%)**	**<.001**	**100 (87.0%)**	**67 (43.8%)**	**<.001**
Reoperation within 30 days after operation (+)	**19 (28.4%)**	**48 (45.3%)**	**.037**	**46 (30.3%)**	**74 (43.8%)**	**.015**	54 (27.0%)	70 (32.7%)	.238	**20 (14.1%)**	**35 (28.2%)**	**.006**	34 (42.5%)	32 (41.6%)	1.000	21 (22.1%)	32 (25.0%)	.637	**40 (34.8%)**	**72 (47.1%)**	**.046**
Surgical site infection other than anastomotic leakage (+)	**6 (9.0 %)**	**31 (29.2%)**	**.001**	**12 (7.9%)**	**30 (17.8%)**	**.012**	**15 (7.5%)**	**44 (20.6%)**	**<.001**	**19 (13.4%)**	**31 (25.0%)**	**.018**	14 (17.5%)	18 (23.4%)	.430	**4 (4.2%)**	**34 (26.6%)**	**<.001**	**19 (16.5%)**	**52 (34.0%)**	**.001**
Anastomotic leakage (+)	15 (22.4%)	38 (35.8%)	.065	**22 (14.5%)**	**53 (31.4%)**	**<.001**	**28 (14.0%)**	**49 (22.9%)**	**.023**	19 (13.4%)	20 (16.1%)	.603	30 (37.5%)	27 (35.1%)	.868	4 (4.2%)	15 (11.7%)	.054	40 (34.8%)	67 (43.8%)	.166
Transfusion ≥5 U (+)	17 (25.4%)	29 (27.4%)	.860	36 (23.7%)	30 (17.8%)	.214	37 (18.5%)	44 (20.6%)	.622	18 (12.7%)	13 (10.5%)	.702	15 (18.8%)	16 (20.8%)	.842	47 (49.5%)	55 (43.0%)	.345	53 (46.1%)	61 (39.9%)	.321
Postoperative unexpected intubation (+)	33 (49.3%)	52 (49.1%)	1.000	**77 (50.7%)**	**52 (30.8%)**	**<.001**	**98 (49.0%)**	**58 (27.1%)**	**<.001**	**32 (22.5%)**	**14 (11.3%)**	**.022**	**34 (42.5%)**	**20 (26.0%)**	**.043**	42 (44.2%)	41 (32.0%)	.070	**56 (48.7%)**	**54 (35.3%)**	**.033**
Postoperative mechanical ventilation ≥48 h (+)	35 (52.2%)	62 (58.5%)	.436	59 (38.8%)	55 (32.5%)	.246	75 (37.5%)	68 (31.8%)	.255	39 (27.5%)	28 (22.6%)	.397	29 (36.3%)	21 (27.3%)	.237	41 (43.2%)	47 (36.7%)	.336	48 41.7%)	61 (39.9%)	.802
Postoperative renal dysfunction (+)	22 (32.8%)	28 (26.4%)	.392	44 (28.9%)	38 (22.5%)	.201	**61 (30.5%)**	**44 (20.6%)**	**.024**	**35 (24.6%)**	**17 (13.7%)**	**.030**	27 (33.8%)	16 (20.8%)	.076	41 (43.2%)	42 (32.8%)	.125	38 (33.0%)	43 (28.1%)	.421
Postoperative central nervous system disorder (+)	0 (0.0%)	1 (0.9%)	1.000	10 (6.6%)	7 ( 4.1%)	.455	8 (4.0%)	3 (1.4%)	.130	**9 (6.3%)**	**1 (0.8%)**	**.022**	7 (8.8%)	2 (2.6%)	.168	1 (1.1%)	0 (0.0%)	.426	3 (2.6%)	5 (3.3%)	1.000
Postoperative cardiac complications (+)	**33 (49.3%)**	**11 (10.4%)**	**<.001**	**63 (41.4%)**	**12 (7.1%)**	**<.001**	**76 (38.0%)**	**12 (5.6%)**	**<.001**	**41 (28.9%)**	**2 (1.6%)**	**<.001**	**38 (47.5%)**	**11 (14.3%)**	**<.001**	**29 (30.5%)**	**7 (5.5%)**	**<.001**	**46 (40.0%)**	**9 (5.9%)**	**<.001**
Postoperative sepsis (+)	34 (50.7%)	47 (44.3%)	.437	55 (36.2%)	50 (29.6%)	.234	62 (31.0%)	70 (32.7%)	.752	**46 (32.4%)**	**26 (21.0%)**	**.039**	33 (41.3%)	22 (28.6%)	.132	25 (26.3%)	45 (35.2%)	.190	40 (34.8%)	66 (43.1%)	.207
Postoperative septic shock (+)	24 (35.8%)	26 (24.5%)	.124	**36 (23.7%)**	**24 (14.2%)**	**.032**	39 (19.5%)	39 (18.2%)	.802	34 (23.9%)	19 (15.3%)	.091	**29 (36.3%)**	**9 (11.7%)**	**<.001**	11 (11.6%)	24 (18.8%)	.192	27 (23.5%)	34 (22.2%)	.883
Postoperative pneumonia (+)	34 (50.7%)	55 (51.9%)	1.000	46 (30.3%)	67 (39.6%)	.081	63 (31.5%)	70 (32.7%)	.833	25 (17.6%)	32 (25.8%)	.134	16 (20.0%)	22 (28.6%)	.264	**9 (9.5%)**	**37 (28.9%)**	**<.001**	27 (23.5%)	42 (27.5%)	.484
Postoperative deep vein thrombosis (+)	0 (0.0%)	0 (0.0%)		3 (2.0%)	5 (3.0%)	.726	3 (1.5%)	2 (0.9%)	.676	5 (3.5%)	0 (0.0%)	.063	4 (5.0%)	1 (1.3%)	.367	3 (3.2%)	1 (0.8%)	.315	2 (1.7%)	2 (1.3%)	1.000
Postoperative pulmonary embolism (+)	0 (0.0%)	0 (0.0%)		2 (1.3%)	3 (1.8%)	1.000	4 (2.0%)	2 (0.9%)	.163	2 (1.4%)	0 (0.0%)	.500	**6 (7.5%)**	**0 (0.0%)**	**.028**	0 (0.0%)	1 (0.8%)	1.000	5 (4.3%)	1 (0.7%)	.087
Postoperative urinary tract infection (+)	1 (1.5%)	8 (7.5%)	.156	**0 (0.0%)**	**8 (4.7%)**	**.008**	4 (2.0%)	9 (4.2%)	.263	**4 (2.8%)**	**12 (9.7%)**	**.021**	2 (2.5%)	8 (10.4%)	.053	5 (5.3%)	6 (4.7%)	1.000	3 (2.6%)	10 (6.5%)	.162
Postoperative pancreatic fistula (+)				11 (7.2%)	21 (12.4%)	.138	10 (5.0%)	13 (6.1%)	.673										48 (41.7%)	72 (47.1%)	.457
Postoperative pancreatic fistula ≥grade C (+)				4 (2.6%)	10 (5.9%)	.179	6 (3.0%)	8 (3.7%)	.789										29 (25.2%)	39 (25.5%)	1.000
Postoperative biliary fistula (+)																8 (8.4%)	34 (26.6%)	.001			

ASA2, American Society of Anesthesiologists Classification 2.

The significance of bolded terms was “*P* < 0.05”.

## 4 DISCUSSION

We found that the 30‐day mortality, which was a standard QI for international comparisons, was not sufficient as a QI for all gastrointestinal cancer surgeries. Surgical stress, risk of complications, surgery‐related mortality rate, and the 30‐day mortality differ depending on the type of surgical procedure. Even when the type of surgical procedure was identical, the risk of surgery‐related deaths and complications varied depending on patient‐related risks, such as age and comorbidities.^12^, ^13^, ^14^ However, from an overall perspective, the risk roughly reflects the difference associated with the type of surgical procedure. Also, in our data, surgery‐related mortality rates varied depending on the type of surgical procedure, where highest values were found in hepatectomies and lowest in low anterior resections. Previous reports, based on a comparison of 30‐day mortality rates, have shown that surgical outcomes were more favorable in Japan than in Europe and the USA.^15^

The number of surgeries carried out, rate of laparoscopic surgeries, length of hospital stay, surgery‐related mortality rates, and rate of complications have been used as indicators for the evaluation of surgical outcomes, or qualities, in individual facilities, and have been used for comparisons between facilities in terms of outcomes (qualities). However, the number of surgeries carried out by each facility, as well as the length of hospital stay, varies depending on each country's health‐care system. In addition, for the rate of complications, such as postoperative surgical site infections and anastomotic leakage, the capture ratio may differ depending on diagnostic criteria and postoperative care systems, which makes it difficult to carry out a comparison of true incidences. Safety is the most basic requirement for carrying out surgery, and treatment outcomes should be evaluated upon securing a certain safety level. From our perspective, surgery‐related mortality rates are believed to be of utmost importance as indicators for the evaluation of surgical quality.

In Japan, the length of postoperative hospital stays are relatively long and, as a result, the capture ratio is high for postoperative complications, including those that are minor and delayed. Cases of hospital transfer or hospital discharge without alleviation of complications are few, and this could be the reason why surgery‐related mortalities nearly match in‐hospital mortalities. Meanwhile, in Europe and the USA, duration of postoperative hospital stay is often short, and surgery‐related deaths among in‐hospital deaths are difficult to determine. Similarly, 30‐day mortality rates do not reflect all aspects of surgical outcomes or qualities.

The findings of the present study show that postoperative complications categorized as Clavien‐Dindo classification grade III or greater, postoperative complications categorized as Clavien‐Dindo classification grade IV or greater, unplanned postoperative intubations, and cardiac complications are factors with significantly high percentages of mortality within 30 days, irrespective of the type of surgical procedure, and factors with low association with surgical procedures, such as renal dysfunction (distal gastrectomy, right hemicolectomy), central nervous system disorder (right hemicolectomy), sepsis (right hemicolectomy), and septic shock (total gastrectomy, low anterior resection) were also extracted. In contrast, our results also indicated that factors highly associated with the type of surgical procedure, such as reoperation within 30 days and anastomotic leakage, had significantly lower percentages of deaths occurring within 30 days after surgery. Our data show that factors linked to surgery‐related deaths differ depending on the type of surgical procedure, and that when the 30‐day mortality rate was used as a QI for an evaluation of surgery outcome or quality, the capture ratio for the determination of deaths associated with surgical technique‐related complications may be low or otherwise useless, depending on the respective surgical procedure. Furthermore, our data suggest that when the rate of surgical site infections (except those as a result of anastomotic leakage) is high in surgeries other than low anterior resection, the surgery‐related mortality rate is likely to be high even when the 30‐day mortality rate is low. Also, when the rate of anastomotic leakage is high in esophagectomy, total gastrectomy, and distal gastrectomy, the surgery‐related mortality rate is likely to be high, even when the 30‐day mortality rate is low. Similarly, in esophagectomy, right hemicolectomy, pancreaticoduodenectomy, the surgery‐related mortality rate is likely to be high when the rate of reoperation within 30 days is high, even when the 30‐day mortality rate is low, and in hepatectomies with large amounts of bile leakage, surgery‐related mortality rate is likely to be high, even when the 30‐day mortality rate is low and, as a result, surgery‐related mortality may be difficult to evaluate properly.

Meanwhile, in low anterior resections, surgery‐related mortalities can be evaluated properly through evaluation of the 30‐day mortality rate. Thus, in order to evaluate the outcomes or qualities of surgery, the 30‐day mortality rate and other indicators, such as complications, will need to be assessed in combination with outcomes or qualities of surgery. For example, the incidence of anastomotic leakage in total gastrectomy and distal gastrectomy or bile leakage in hepatectomy may be useful. And to use these indicators could fit the actual clinical feelings of surgeons. A comparison with worldwide outcomes or qualities of surgery, or benchmarking, will be necessary to improve the outcomes or qualities of surgery in Japan.^16^ However, data regarding Japan, in which surgery‐related mortality is well determined, were based on the Japanese health‐care system, and it remains unknown whether these observations are unique to Japan or are universal and shared worldwide. Our results, which were derived from data collected in Japan, could be used to guide an evaluation of their association with medical circumstances in all countries worldwide, through an international endeavor.

## 5 CONCLUSION

The 30‐day mortality rate is definitely useful as a QI for the evaluation of the outcomes or qualities of gastrointestinal cancer surgeries, but it is not necessarily sufficient to cover all types of surgical procedures. Depending on the type of surgical procedure, evaluations of surgical outcome (or qualities) may need to be carried out in combination with the use of the 30‐day mortality rate and other indicators, such as complications.

## DISCLOSURE

Funding: Sources of funding for research and/or publication: The Japanese Society of Gastroenterological Surgery.

Conflicts of Interest: Hiroaki Miyata and Hiroyuki Yamamoto are affiliated with the Department of Healthcare Quality Assessment at the University of Tokyo, and the department is endowed by Johnson & Johnson K.K., Nipro Co., Teijin Pharma Ltd, Kaketsuken K.K., St. Jude Medical Japan Co., Ltd, Novartis Pharma K.K., Taiho Pharmaceutical Co., Ltd, W. L. Gore & Associates, Co., Ltd, Olympus Corporation, and Chugai Pharmaceutical Co., Ltd. None of the organizations had any role in design and conduct of the study, data collection, data analysis, data management, data interpretation, or the preparation, review, approval of this manuscript. The other authors declare that they have no conflicts of interest.

Author Contribution: Substantial contributions to conception and design, or acquisition of data, or analysis and interpretation of data; T.M., H.Y., S.M., K.K., G.W., H.M., Y.D., M.M. Drafting the article or revising it critically for important intellectual content; T.M., H.Y., S.M., K.K., G.W., H.M. Final approval of the version to be published; Y.S., Y.D., M.M.
